# Phosphatase of regenerating liver-3 inhibits invasiveness and proliferation in non-small cell lung cancer by regulating the epithelial-mesenchymal transition

**DOI:** 10.18632/oncotarget.7985

**Published:** 2016-03-08

**Authors:** Sheng-Yi Lin, Yue-Xun Lee, Sung-Liang Yu, Gee-Chen Chang, Jeremy J.W. Chen

**Affiliations:** ^1^ Institute of Biomedical Sciences, National Chung Hsing University, Taichung, Taiwan; ^2^ Agricultural Biotechnology Center, National Chung Hsing University, Taichung, Taiwan; ^3^ Department of Clinical Laboratory Sciences and Medical Biotechnology, National Taiwan University College of Medicine, Taipei, Taiwan; ^4^ Division of Chest Medicine, Department of Internal Medicine, Taichung Veterans General Hospital, Taichung, Taiwan

**Keywords:** PRL-3, NSCLC, invasion, migration, tumor growth

## Abstract

Phosphatase of regenerating liver-3 (PRL-3) has been reported to be associated with colon and gastric cancer metastasis. However, the role and function of PRL-3 in human non-small cell lung cancer cells is unknown. Our studies showed that the expression of *PRL-3*mRNA and protein are higher in less invasive human lung adenocarcinoma cells than in highly invasive cell lines. Ectopic expression of PRL-3 reduced cell capacity for anchorage-dependent growth, anchorage-independent growth, migration, and invasion *in vitro*, as well as tumorigenesis *in vivo*. Conversely, catalytic (C104S) and prenylation-site (C170S) mutants enhanced cell invasion. Microarray profiling of PRL-3 transfectants revealed the pathways potentially involving PRL-3, including the epithelial-mesenchymal transition (EMT), extracellular matrix remodeling, and the WNT signaling pathway. Furthermore, we demonstrated that increased PRL-3 reduced Slug and enhanced E-cadherin gene expression through the AKT/GSK3β/β-catenin pathway. In conclusion, our data suggest that PRL-3 might play a tumor suppressor role in lung cancer, distinct from other cancers, by inhibiting EMT-related pathways.

## INTRODUCTION

Non-small-cell lung cancer (NSCLC) is responsible for the most frequent cause of cancer-related death [1–3]. Although approximately 30% of patients with NSCLC are diagnosed at an early stage and receive therapy, the tumor typically recurs within 5 years with metastasis [3–6]. Metastasis is one of the major determinants leading to cancer progression and a complex and multi-step process including enhanced cellular motility and extracellular matrix degradation [7].

Protein tyrosine phosphorylation is an important posttranslational modification regulating the intracellular signaling pathways that determine cellular physiologic and pathologic processes. Phosphorylation is regulated by protein tyrosine phosphatases (PTPs) and protein tyrosine kinases (PTKs) [8]. Phosphatase of regenerating liver-3 (PRL-3), also known as PTP4A3, is a PRL-PTP identified as possessing a C-terminal prenylation motif (CAAX box). This motif causes PRL-3 to be posttranslationally modified by farnesyltransferase, and farnesylated PRL-3 localizes to the plasma membrane and early endosomes. Inhibiting farnesylation leads to a localization shift of PRL-3 into the nucleus to mediate its function. The catalytic site CX5R of PRL-3 functions as a dual-specificity phosphatase that is able to dephosphorylate tyrosine, serine, and threonine residues in some cases [9].

The functional role of PRL-3 in cancer metastasis is rather conflicting and controversial. For example, PRL-3 is consistently highly expressed in metastatic colorectal tumors compared with non-metastatic tumors and the normal colorectal epithelia [10], and overexpression of PRL-3 in malignant human myeloma cells may alter their aggressiveness and migration [11]. Several studies have also indicated that PRL-3 may serve as a biomarker for poor prognosis in gastric cancer, ovarian cancer, breast cancer, and colon cancer [12–14]. Although elevated PRL-3 expression appears to have an important role in the enhanced metastatic potential of tumor cells, a recent report showed that PRL-3 gene expression is down-regulated 10-fold in metastatic lung cancer compared with normal lung tissue [15]. These results indicate that PRL-3 might play different roles in various carcinomas. However, the role of PRL-3 in NSCLC has remained unclear with respect to the PRL-3-mediated cellular signaling pathways or the cellular substrates of PRL-3.

The identification of signaling pathways involving PRL-3 is key to uncovering its roles in cancer progression. In this study, we investigated the cellular functions and mechanism of action of PRL-3 in NSCLC using *in vitro*, *in vivo*, and functional genomic approaches. Our results may help to clarify the multifunctional role of PRL-3 and add new insights into tumor biology.

## RESULTS

### PRL-3 expression in lung cancer cell lines with different invasiveness

**Figure 1:**
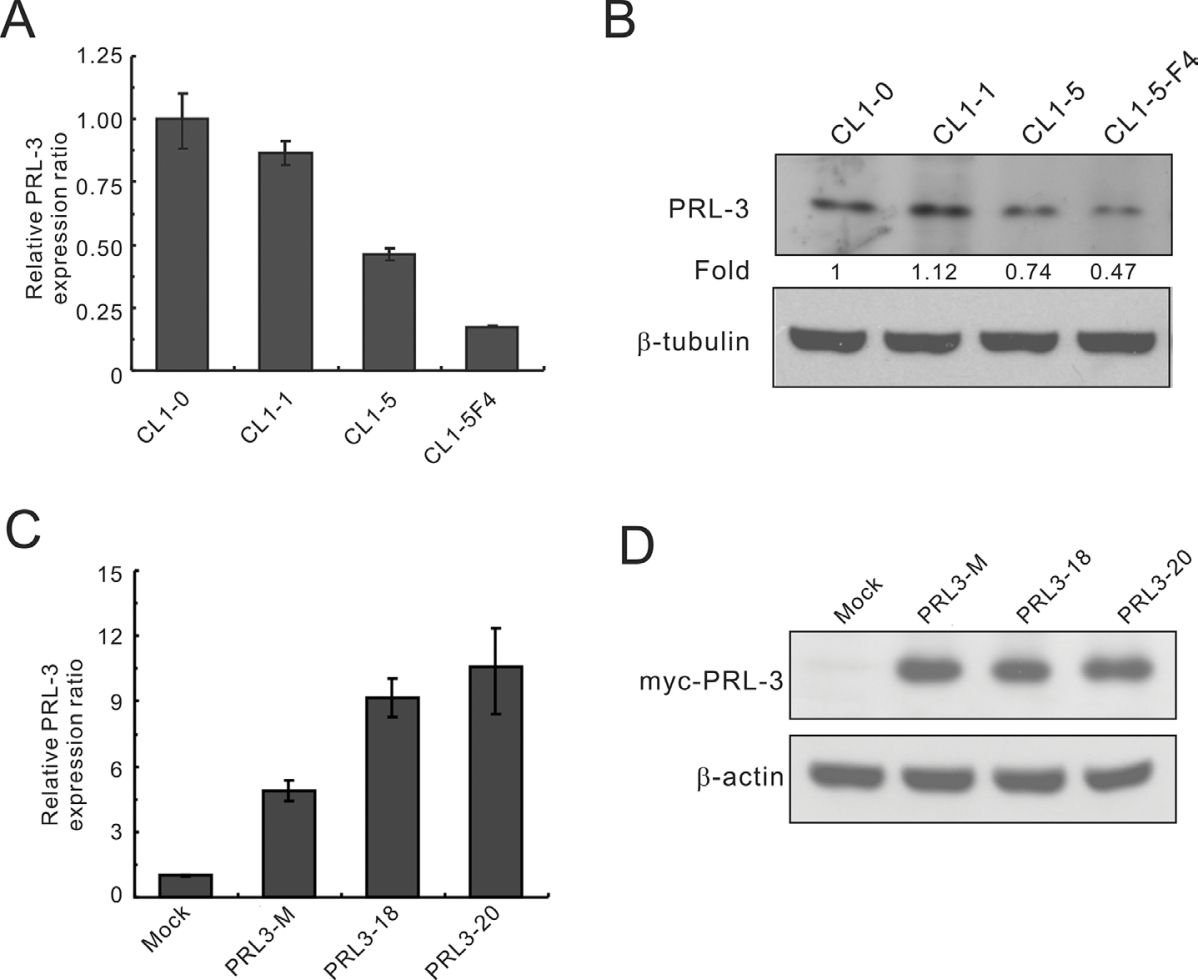
PRL-3 expression in lung cancer cell lines with increasing invasiveness and transfectants The invasive ability of the cell lines is as follows: CL1-0 < CL1-1 < CL1-5 < CL1-5F4. (**A**) *PRL-3* mRNA expression levels in cell lines, as measured by real-time RT-PCR. (**B**) PRL-3 protein levels, as detected by Western blot analysis. β-tubulin was used as an internal control. (**C**) *PRL-3* mRNA expression in the transfectants, as measured by real-time RT-PCR. CL1-5 cells were transfected with *pCMV-Tag3B-PRL-3* or vector alone to establish stable cell clones, including mock, mixed, and single cell clones. TBP was used as an internal control. (**D**) PRL-3 protein expression in transfectants, as measured by Western blot analysis. β-tubulin was used as a loading control. Real-time RT-PCR was assessed in triplicate.

To evaluate the potential role of PRL-3 in lung cancer, we first analyzed its expression in lung cancer cell lines with various invasive abilities using real-time RT-PCR and Western blot analysis. Real-time RT-PCR analysis showed that *PRL-3* mRNA expression was higher in CL1-0 and CL1-1 lung cancer cells with less invasive activity than in CL1-5 and CL1-5-F4 cells with highly invasive activity (Figure 1A). PRL-3 protein levels by Western blot analysis were also higher in the CL1-0 and CL1-1 than in CL1-5 and CL1-5-F4 (Figure 1B). The data revealed a correlation between PRL-3 expression and cell invasive capability in NSCLCs. To investigate the cellular functions of PRL-3, we generated stable CL1-5 cell lines expressing Myc-tagged PRL3 or empty vector. We examined PRL-3 expression in transfectants using real-time RT-PCR and Western blot assays (Figure 1C and 1D). It is evident that either a mixed clone or single clones (PRL3-18 and PRL3-20) expressed a higher level of PRL-3 transcription and translation products than the mock control.

### Identification of the sub-cellular distribution of PRL-3 and mutant forms

**Figure 2:**
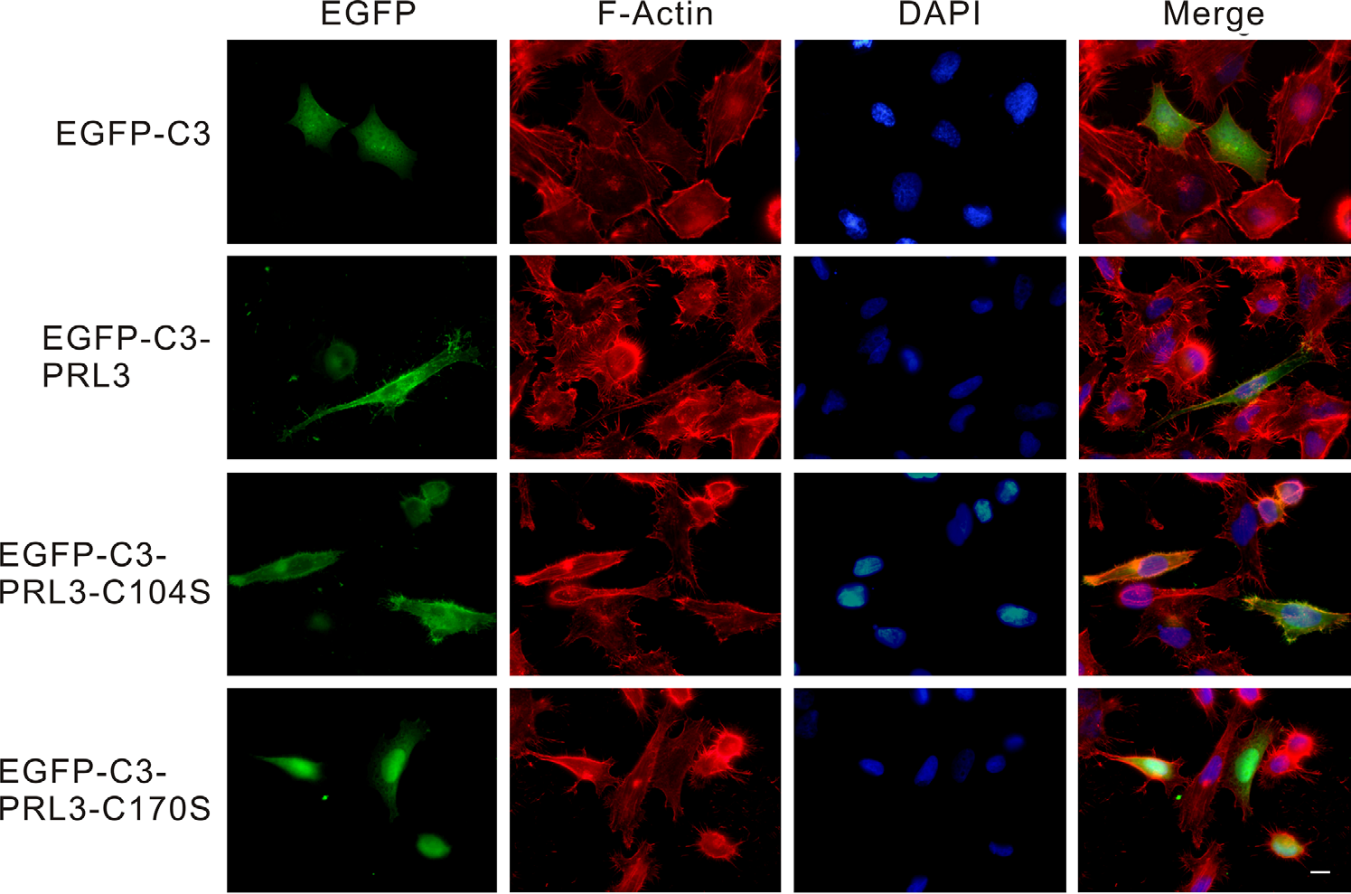
The subcellular distribution of wild-type and mutant PRL-3 in CL1-5 cells EGFP-tagged wild-type and mutant (C104S and C170S) PRL-3, as well as EGFP-C3 mock were transiently transfected into CL1-5 cells and detected by fluorescent microscopy. Green: wild-type or mutant EGFP-C3-PRL3 fusion protein; red: F-actin staining by phalloidin; blue: nuclear DNA staining by DAPI. Scale bars, 10 μm.

To identify the sub-cellular localization of wild-type PRL-3, a phosphatase-dead mutant form (PRL3/C104S) and a prenylation-site mutant form (PRL3/C170S) or the EGFP-tagged PRL3 was transiently transfected into CL1-5 cells and then observed under a fluorescence microscope. In the *pEGFP-C3*-transfected cells, green fluorescence was evenly distributed throughout the whole cell; however, the fluorescence was mostly located at the plasma membrane in the *pEGFP-C3-PRL3*- and *pEGFP-C3-PRL3-C104S*-transfected cells and mainly located in the nucleus and cytoplasm in the *pEGFP-C3-PRL3-C170S*-transfected cells (Figure 2).

### PRL-3 overexpression reduces cell invasion and migration

**Figure 3:**
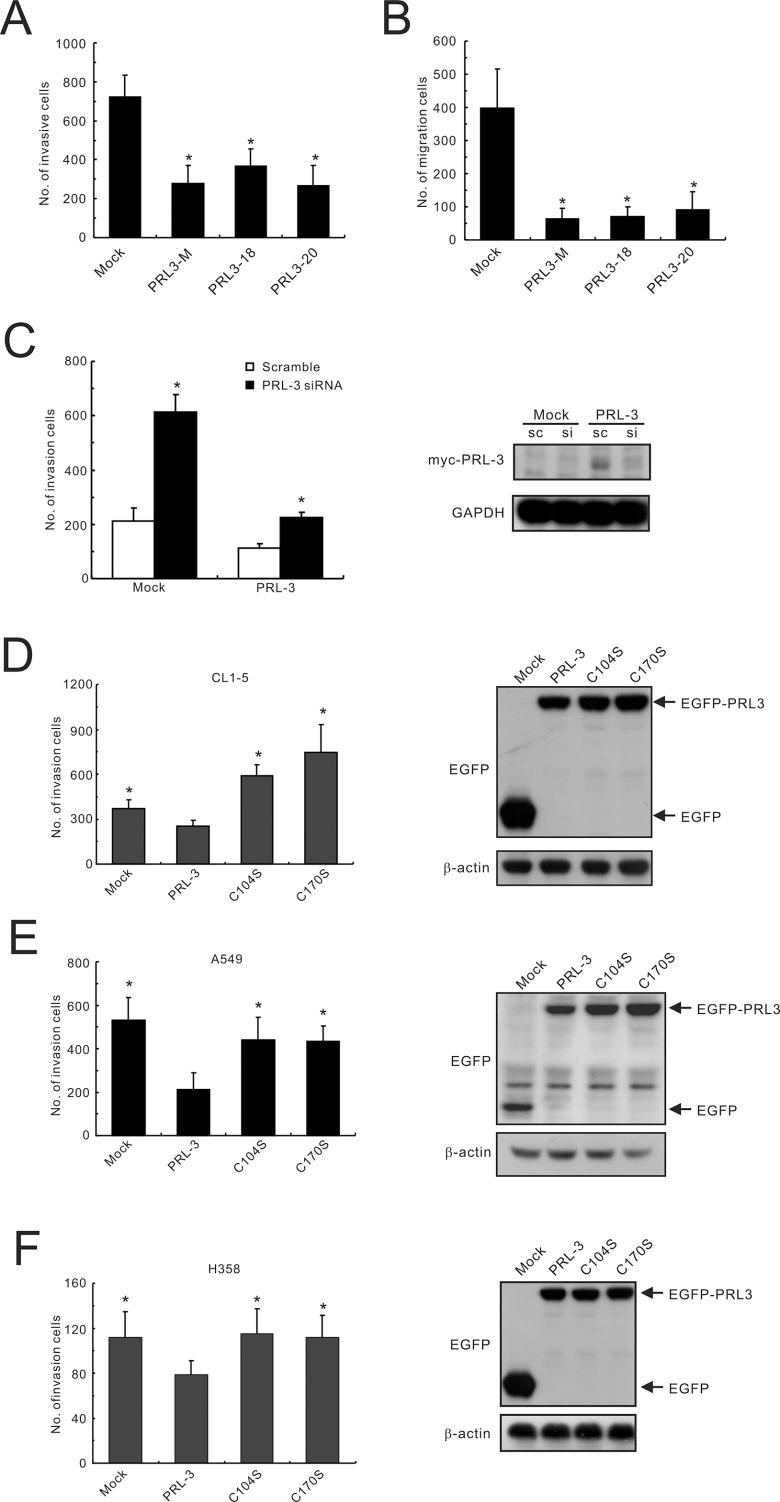
Inhibition of lung cancer cell invasion and migration by PRL-3 expression (**A**) Invasiveness and (**B**) migration ability of PRL-3 transfectants, as determined by a transwell apparatus. Mixed: mixed cell clones; Single: single cell clones PRL3-18 and PRL3-20); Mock; vector alone. The data are presented as the mean ± S.D. of three experiments. *P* values are compared with mock. (**C**) Invasive ability of lung cancer cells with PRL-3 knockdown. PRL-3 was silenced by a small-interfering RNA (siRNA) in PRL-3-overexpressing CL1-5 cells (PRL-3) and control cells (Mock), and then subjected to an *in vitro* cell invasion assay. Right panel: PRL-3 expression level after silencing. The data are expressed as the mean ± S.D. of three experiments, and *P* values are compared with scrambled siRNA control cells. (**D**–**F**) The effect of transient expression of wild-type PRL-3 and mutants PRL-3/C104S and PRL-3/C170S on cell invasive properties. The tested lung cancer cell lines include CL1-5 (D), A549 (E), and H358 (F). The ectopic expression levels of PRL-3 protein are displayed in the right panels, as detected by Western blotting. The data are presented as the mean ± S.D. of three experiments. *P < 0.05, compared with wild-type PRL-3 cells.

With regard to cell function, the induction of PRL-3 expression (PRL3-mixed, PRL3-18, and PRL3-20) led to a significant reduction (approximately 2-3-fold) in invasive activity in the Matrigel invasion assay compared with that of mock-transfected cells (Mock; Figure 3A). The migration capabilities of PRL3-expressing cells were also lower than those of the control cell (reduced by 4.4–6.3-fold) in the transwell migration assay (Figure 3B). To confirm the effect of PRL-3 expression on the motility of lung cancer cells, RNA interference technology was employed to examine the cellular capacity for invasion. The results indicated that transient transfection with a PRL3-specific siRNA suppressed protein expression and increased invasion ability in a PRL3-mixed stable cell clone (Figure 3C). Furthermore, transfection with PRL-3-mutant alleles (C104S and C170S) enhanced cell invasion ability in different lung cancer cell lines, including CL1-5, A549, and H358, compared with the PRL-3 wild-type (Figure 3D–3F). However, the result for colorectal adenocarcinoma was quite different from this study but consistent with a previous report [16], indicating that the wild-type PRL-3 enhanced cell invasion in colon cancer SW480 cells compared with mutant PRL-3 and mock control (Supplementary Figure S1).

### PRL-3 inhibits lung cancer cell growth *in vitro* and tumorigenesis *in vivo* and benefits patients' survival

**Figure 4:**
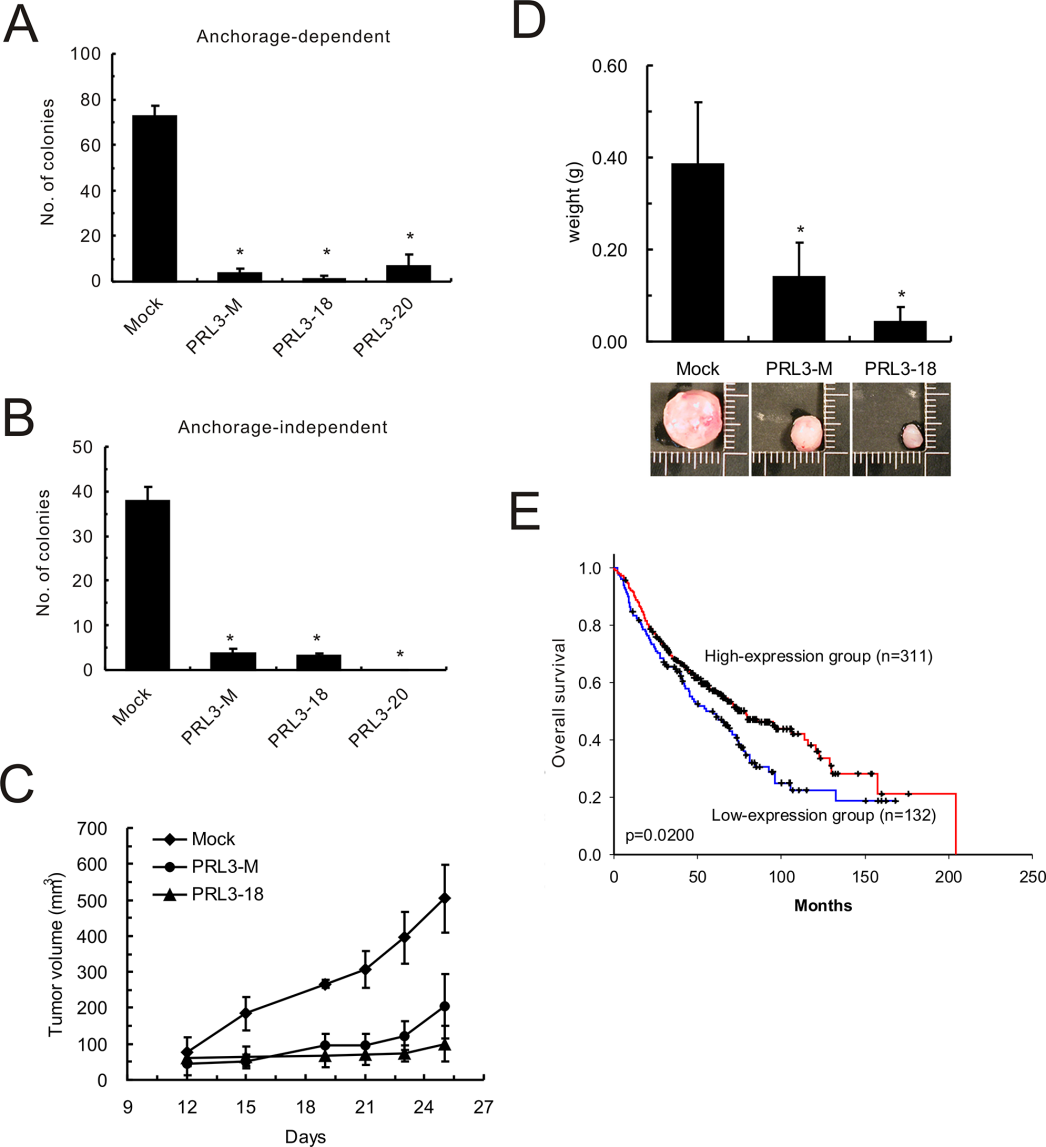
Effect of PRL-3 on lung cancer cell growth, tumorigenesis and patients' survival (**A**) Anchorage-dependent cell growth of PRL-3 transfectants, as measured by colony formation. (**B**) Effect of PRL-3 expression on anchorage-independent growth, as assessed by soft agar assay. Mixed: mixed cell clones; Single: single cell clones PRL3-18 and PRL3-20); Mock: vector alone. The data are presented as the mean ± S.D. of three experiments. *P* < 0.05, compared with control cells (Mock). (**C**) PRL-3 overexpression and reduced tumorigenicity in nude mice. The volumes of tumors derived from control cells (Mock) and PRL-3 expressing cells (PRL3-mixed and PRL3-18) were evaluated at the indicated times. (**D**) The weights of tumors derived from three groups were measured when the nude mice were sacrificed. The columns are presented as the mean ± S.D. (n = 6 mice per group). *P < 0.05, compared with control cells (Mock). (**E**) Decreased PRL-3 expression is correlated with poor survival in NSCLC patients. The probability of overall survival is presented according to *PRL-3* RNA expression level in 443 lung adenocarcinoma patients in a published microarray dataset. The patients were divided into high-expression and low-expression groups using the 70% percentile for the level of PRL-3 RNA as a cut-off point. The overall survival curves were produced by the Kaplan-Meier method, and a 2-tailed log-rank test was used to test the differences between the survival curves.

Proliferation of PRL3-expressing cell lines (PRL3-mixed, PRL3-18, and PRL3-20) was 6-7-fold slower than that of mock cells, as measured by an anchorage-dependent colony-formation assay (Figure 4A). The reduced colony formation effect of PRL3-expressing cells on anchorage-independent growth was indicated by the soft agar assay (Figure 4B). Tumors derived from CL1-5 cells with PRL-3 overexpression grew more slowly than those derived from mock cells in nude mice. The volume of the tumors obtained from the CL1-5/PRL-3 stable clones (PRL3-mixed and PRL3-18) increased to 202 mm^3^ (SD, ± 90.24 mm^3^) and 100 mm^3^ (SD, ± 49.56 mm^3^) 25 days after inoculation, whereas tumors obtained from the mock stable clone increased to 504 mm^3^ (SD, ± 94.98 mm^3^) in nude mice (Figure 4C). The weights of tumors derived from PRL3-expressing cell lines were approximately 0.14 g (PRL3-mixed; SD, ± 0.074 g) and 0.043 g (PRL3-18; SD, ± 0.032 g), respectively, whereas the weight of the tumors derived from vector control cells reached 0.384 g (SD, ± 0.136 g; Figure 4D). Furthermore, to reflect the clinical relevance of PRL-3 in NSCLC patients, we extended our analysis by examining the expression of *PRL-3* mRNA in a large NSCLC patient cohort that had been published previously [17]. Consistent with our *in vitro* and *in vivo* results, the patients with high-level PRL-3 expression had longer overall survival than those with low-level expression (Figure 4E; *P* = 0.02, log-rank test).

### Identification of PRL-3 downstream genes by cDNA microarray analysis

**Figure 5:**
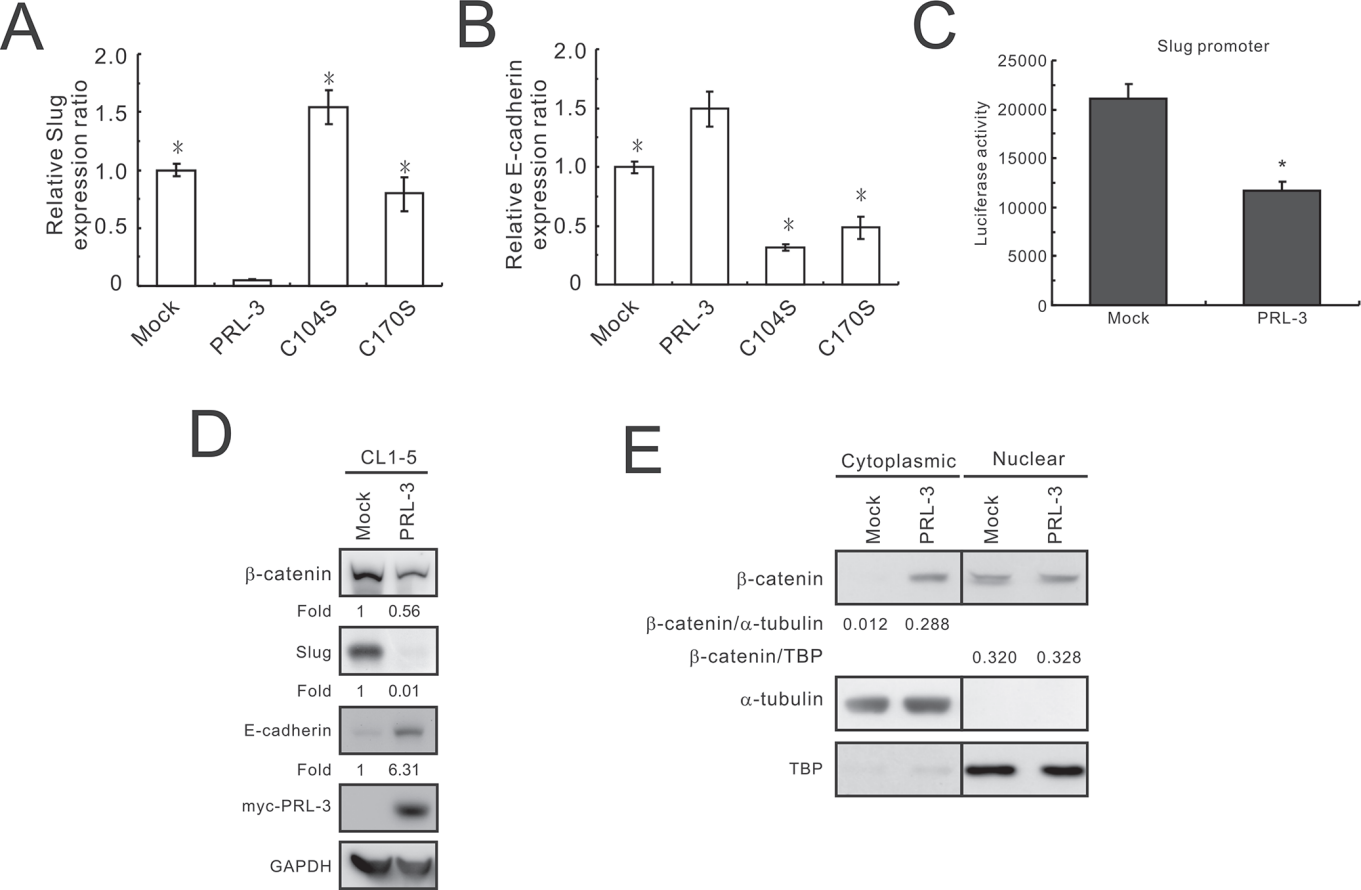
Slug reduction and E-cadherin promotion by PRL-3 overexpression (**A**) Slug and (**B**) E-cadherin expression in wild-type and mutant PRL-3 transfectants, as detected by real-time RT-PCR. Stable CL1-5 transfectants expressing vector alone, wild-type PRL-3, mutant PRL3/C104S, or PRL3/C170S were employed in this study. The data are presented as the mean ± S.D. of triplicates. *P < 0.05, compared with the wild-type PRL-3 cells. (**C**) The effect of PRL-3 expression on Slug promoter activity, as determined by a luciferase reporter assay. CL1-5 cells were transiently co-transfected with the Slug promoter and PRL-3 expression plasmids (PRL-3) or mock control (Mock). The data are shown with the mean ± S.D. of three experiments, and *P* values are compared with control cells (Mock). (**D**) Effect of PRL-3 on β–catenin, Slug, and E-cadherin expression. After seeding for 24 h, cell extracts from CL1-5 cells transfected with PRL-3 or empty vector were analyzed by Western blot analysis. GAPDH was used as an internal control. (**E**) Reduction of nuclear to cytoplasmic ratio of β–catenin by PRL-3 overexpression. Cell extracts were separated into cytoplasmic and nuclear protein fractions and then subjected to Western blot with anti-β–catenin antibody. α–tubulin and TBP served as the loading controls.

Oligonucleotide microarray analysis was used to identify differentially expressed genes between the CL1-5/PRL-3 stable clone and mock control. A total of 931 genes with 2-fold changes in expression levels between the above two transfectants were identified by pathway analysis using MetaCore software. The top 10 signaling pathways identified by MetaCore software are listed in Table 1. Six of these pathways have been shown to affect cell invasion, migration, and apoptosis. Among the affected pathways, the epithelial-to-mesenchymal transition (EMT) pathway attracted our attention. The genes stimulated and suppressed by PRL-3 in the regulation of the EMT pathway are listed in Supplementary Table S2. We found that the invasion-promoting gene *SNAI2* (Snail homolog 2, Slug) was strongly suppressed in PRL-3-expressing CL1-5 cells and that its inhibitory target *CDH1* (E-cadherin) exhibited markedly stimulated expression. To further confirm the effect of PRL-3 on the regulation of Slug and E-cadherin, the cells transfected with PRL-3 wild-type and mutant (C104S and C170S) constructs were used to measure the mRNA expression using SYBR Green and real-time RT-PCR. *Slug* mRNA levels were down-regulated in PRL-3 wild-type cells and elevated in PRL-3-mutant cells (Figure 5A), whereas the mRNA level of E-cadherin was up-regulated by wild-type PRL-3 and reduced by mutant PRL-3 expression (Figure 5B).

**Table 1:** The top 10 signaling pathways affected by PRL-3 overexpression

Ranking	Pathway	*P*
1	Development_Regulation of epithelial-to-mesenchymal transition (EMT)	7.38E-08
2	Development_TGF-beta-dependent induction of EMT via SMADs	9.48E-07
3	Cell adhesion_ECM remodeling	4.14E-06
4	Immune response_Antiviral actions of Interferons	4.14E-06
5	Development_WNT signaling pathway	4.96E-06
6	Transport_RAB3 regulation pathway	4.81E-05
7	Apoptosis and survival_Regulation of Apoptosis by Mitochondrial Proteins	6.15E-05
8	Signal transduction_Calcium signaling	7.01E-05
9	Stimulation of TGF-beta signaling in lung cancer	1.13E-04
10	Main pathways of Schwann cells transformation in neurofibromatosis type 1	1.29E-04

To further examine the effect of PRL-3 on *Slug* transcriptional regulation, we used a luciferase reporter assay to determine the *Slug* promoter activity. *Slug* promoter activity was markedly reduced by PRL-3 compared with the mock control (*p* = 0.03; Figure 5C). Western blot analysis also showed that Slug expression was dramatically decreased and E-cadherin was significantly increased in the wild-type PRL-3-overexpressing cells compared with the mock control (Figure 5D). In addition, the protein level of β-catenin was diminished in the PRL-3 transfectant. Furthermore, we also found that the nuclear to cytoplasmic ratio of β-catenin is decreased when cancer cells overexpress PRL-3, from 26.67 in the Mock down to 1.14 in the PRL-3 transfectant (Figure 5E).

### PRL-3 suppresses Slug expression via the AKT-GSK3β pathway

**Figure 6:**
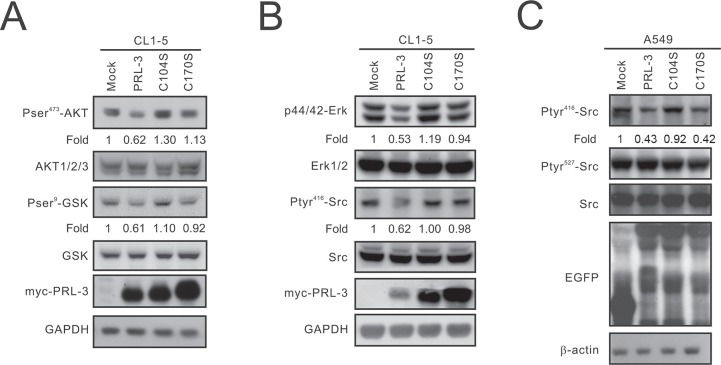
The inhibitory effect of PRL-3 on the AKT-GSK3β pathway and SRC and ERK1/2 activation (**A**) Reduction of the phosphorylation of AKT and GSK3β by PRL-3. Cell lysates were analyzed by Western blot analysis with antibodies against pAKT473, AKT1/2/3, pGSKβ9, GSKβ, and the c-myc tag. GAPDH was used as an internal control. (**B**) Reduction of SRC activation and ERK1/2 phosphorylation by PRL-3. The expression levels of pERK42/44, ERK1/2, pSRC416, SRC, and myc-tagged PRL-3 in wild-type and mutant PRL-3 stable clones were detected by Western blotting. β-actin was used as a loading control. (**C**) Suppression of SRC phosphorylation in A549 cells by PRL-3. Cell lysates from A549 cells transiently expressing wild-type and mutant alleles of PRL-3 were analyzed by Western blot analysis with anti-pSRC416, anti-pSRC527, anti-SRC, and anti-EGFP. β-actin was used as a loading control.

Previous studies have demonstrated that Slug is increased by β-catenin [18]. Therefore, we focused on the mechanism by which PRL-3 regulates the β-catenin-Slug axis. PRL-3 is a member of the PTP family, and its phosphatase activity may regulate protein phosphorylation. Our data revealed that AKT phosphorylation is decreased in PRL-3-expressing cells compared with that of the mock control as well as PRL-3/C104S and PRL-3/C170S mutant cells. Moreover, overexpression of wild-type PRL-3 led to a decrease in GSK3β phosphorylation (Figure 6A).

### PRL-3 overexpression leads to a reduced phosphorylation of ERK1/2 and SRC

SRC is a well-known oncogene that closely correlates with cell proliferation and invasion [19]. A previous report indicated that SRC could be activated by PRL-3 [20]. In this study, we examined the phosphorylation of SRC and its downstream target, ERK1/2. Interestingly, our results showed that wild-type PRL-3 decreased the phosphorylation of SRC and ERK1/2 in CL1-5 cells, while the C104S and C170S PRL-3 mutants exhibited recovered phosphorylation (Figure 6B). We also transiently transfected the EGFP-tagged PRL3, C104S, and C170S constructs into A549 cells and found that pSRC416 phosphorylation, but not pSRC527 phosphorylation, was decreased (Figure 6C).

## DISCUSSION

Previous studies have shown that PRL-3 expression positively correlates with cancer progression, especially in colorectal cancer [21, 22]. However, a clinical investigation in lung cancer revealed that PRL-3 is down-regulated in metastatic lesions compared with primary tumors or normal lung [15]. To understand the true role of PRL-3 in lung cancer, *in vitro* and *in vivo* approaches were employed in this study. We found that PRL-3 overexpression reduced lung cancer cell growth, migration, and invasion *in vitro* as well as tumorigenesis in nude mice. The transcriptomic analysis indicated that the pathways involved in the cell functions affected by PRL-3 include the regulation of EMT, TGF-β-dependent induction of EMT via SMADs, cell adhesion, and the Wnt signaling pathway. Further investigations revealed that the inhibitory effect of PRL-3 on lung cancer cell motility may occur by suppressing the phosphorylation of AKT and GSK3β, which further decreases Slug and increases E-cadherin expression.

A number of studies have indicated that PRL-3 is highly expressed in several types of cancer [23–25] and is expressed to a greater extent in metastatic lesions than in primary cancers [25–27]. Nevertheless, there are few conflicting studies that have described the correlation between PRL-3 expression and lung cancer progression. Two reports showed no PRL-3 increase in lung cancer tissues compared with normal ones [15, 28], whereas another showed that PRL-3 is overexpressed in NSCLC and correlated with clinical stage [29]. However, our data confirmed the role of PRL-3 in lung cancer and indicated that PRL-3 expression negatively correlates with lung cancer cell motility and growth (Figures 3 and 4). In contrast with lung cancer, ectopic expression of wild-type PRL-3 increased cell invasion in colon cancer cell line SW480 (Supplementary Figure S1), which is consistent with the previous report. [16].

In addition to the *in silico* clinical investigation of PRL-3 (Figure 4E), we also analyzed the immunohistochemistry data from the human lung and colorectal cancer specimens published in the Human Protein Atlas database (www.proteinatlas.org), which is a tissue-based map of the human proteome [30]. These data highlight the differential expression of PRL-3 between the lung and colorectal cancer. PRL-3 was not detected in any of the clinical lung cancer samples assessed (*n* = 12), whereas PRL-3 was detected in 9 of 10 samples in the colorectal cancer specimens (from low- to high-level expression; Supplementary Figure S2). In summary, these studies led us to speculate about a different role and mechanism for PRL-3 in lung cancer from the other types of cancer.

Because unprenylated PRL-3 is localized to the nucleus and loses its function in tumor metastasis [31], an unprenylated mutant (PRL-3/C170S) was used to explore the role in the process of tumor cell metastasis in this study. In addition, PRL-3 with C104S mutation in the catalytic site loses biological function [32], which could reveal the effect of catalytic functionality on cell invasion. Our data showed that wild-type PRL-3 inhibits cell invasion in several lung cancer cell lines, but the C104S and C170S variants enhance invasiveness. The results indicated that PRL-3 requires prenylation and catalytic activity to inhibit the process of cell metastasis in lung cancer cells. However, the results are vastly different from those for colon cancer (Supplementary Figure S1).

Microarray analysis showed that the pathway most affected by PRL-3 is EMT, in which the relationship between Slug and E-cadherin is well known to involve cancer cell motility and metastasis [33, 34]. Our findings demonstrated that increased PRL-3 can significantly reduce Slug and enhance E-cadherin expression. Interestingly, a recent study revealed that PRL-3 overexpression in salivary adenoid cystic carcinoma cell line can promote Slug and down-regulate E-cadherin, leading to increased cell motility [35]. This distinct relationship between PRL-3 and these targets demonstrates the multiple faces of PRL-3 in different cancer types. Recently, active AKT has been implicated in the stabilization of β-catenin through the phosphorylation of GSK-3β, further leading to the transactivation of Slug, which was increased by β-catenin in endometrial cancer cell lines [36]. In our study, PRL-3 overexpression suppressed β-catenin protein expression, AKT activation, and GSK3β phosphorylation. Therefore, we propose that PRL-3 may suppress Slug through the AKT-GSK3β-catenin pathway.

In addition to Slug, we found that some genes previously reported as oncogenes, including angiopoietin-2 [37, 38] and *DKK1* [39, 40], are also suppressed by PRL-3 (Supplementary Figure S3A and S3B). Angiopoietin-2, an angiogenic regulator, promotes MCF7 cell survival through ILK-AKT1/2 signaling and dramatically decreases lung cancer patients' survival [37, 38]. Our data showed that PRL-3 overexpression reduces the phosphorylation of AKT and the mRNA expression of Angiopoietin-2, which is rescued by the PRL-3 mutants. DKK1 is an inhibitor of the Wnt/β-catenin pathway. A recent study reported that DKK1 can suppress the progression of colon cancer [40] but elevates the invasion capacity of esophageal carcinoma cell line EC-9706 [39] and diminishes the survival of lung cancer patients [41]. These studies indicated that the role of DKK1, just like PRL-3, is different in various cancers. Moreover, our data also showed that PRL-3 can stimulate certain tumor suppressor genes, such as A-Kinase anchor protein 12 (*AKAP12*). AKAP12 is an important regulator of the β_2_-adrenergic receptor complex, which controls cell signaling, cell adhesion, mitogenesis, and differentiation [42]. AKAP12 has been associated with certain human cancers, including lung carcinoma [43] and hepatocellular carcinoma [44]. Our real-time RT-PCR results indicated that wild-type PRL-3 stimulates the mRNA expression of *AKAP12*, whereas mutant-PRL-3 returns the mRNA expression to normal levels (Supplementary Figure S3C). These results suggest that PRL-3 may act as a tumor suppressor in lung cancer.

**Figure 7:**
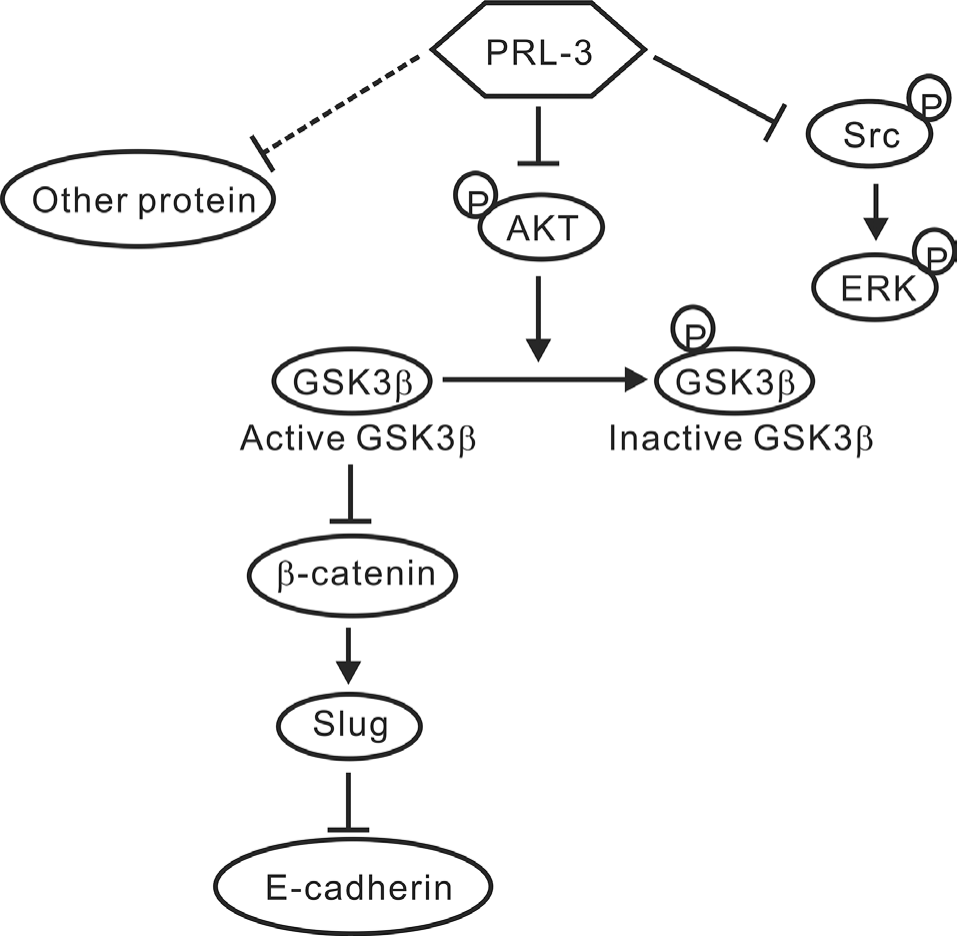
A proposed model of PRL-3-mediated suppression in lung cancer The schematic showed that PRL-3 inhibited cell invasion and migration through suppressing Slug expression via the reduced AKT phosphorylation and GSK3β signaling. PRL-3 overexpression would decrease AKT phosphorylation at Ser^473^, and in turn, the dephosphorylated AKT reduces the phosphorylation of GSK3β and enhances GSK3β activity, leading to the down-regulation of β-catenin and Slug, and the up-regulation of E-cadherin. Enhanced PRL-3 expression also inhibited SRC and ERK phosphorylation, which may lead to the inhibition of lung cancer cell proliferation.

In conclusion, PRL-3 exhibited the characteristics of a tumor suppressor in NSCLC, and the phosphatase's inhibitory effect on lung cancer progression might occur through the down-regulation of Slug expression via the AKT-GSK3β pathway, further leading to increases in E-cadherin. However, we cannot exclude the possibility that other proteins or pathways involved in cancer metastasis and tumorigenesis are also modulated by PRL-3 (Figure 7). These efforts may help researchers understand the multifaceted role of PRL-3 in tumor biology and clarify the actual role of PRL-3 in NSCLC from other cancer types.

## MATERIALS AND METHODS

### Cell culture

The human lung adenocarcinoma cell lines, CL1-0, CL1-1, CL1-5, and CL1-5-F4 [45], with different invasive capacities, as well as A549, H358, and the human colon adenocarcinoma cell line SW480 (American Type Culture Collection, ATCC, Rockville, MA, USA) were cultured at 37°C in a 5% CO_2_ humidified atmosphere. Cells were maintained in RPMI 1640 (GIBCO BRL, Grand Island, NY, USA) with 10% heat-inactivated fetal bovine serum (GIBCO BRL) and 1% penicillin and streptomycin (GIBCO BRL).

### Construction of expression vectors and stable transfection

To create the PRL-3 expression constructs, the PRL-3 coding region was amplified by PCR using the forward primer 5′-CGGGATCCGCTCGGATGAACCGCC-3′, which introduced a BamHI site, and the reverse primer 5′-CCGCTCGAGCTACATAACGCAGCACCGGGTC-3′, which introduced an XhoI site. Full-length *PRL-3* cDNA was inserted into *pCMV-Tag3B* (Stratagene, La Jolla, CA, USA), *pEGFP-C3* vector (Clontech Laboratories, Inc., Palo Alto, CA, USA). The inactive PRL-3 mutant constructs (C104S and C170S) [16, 46] were produced with the QuikChange site-directed mutagenesis kit (Stratagene). All constructs were confirmed by DNA sequencing.

CL1-5 cells expressing a low level of PRL-3 were transfected with *pCMV-Tag3B-PRL3* or *pCMV-Tag3B* empty vector using Lipofectamine 2000 reagent (Invitrogen Life Technologies, Carlsbad, CA, USA), according to the manufacturer's protocol. After culturing in a medium containing 400 μg/ml of Geneticin (G418; GIBCO BRL) for 2–3 weeks, single-cell clones were isolated. For silencing PRL-3 expression, cells were transfected with a PRL3-specific siRNA (cat no. s22005, *Ambion* Biosystems, Austin, TX, USA) or scrambled siRNA (cat no. *AM4611, Ambion Biosystems*) using the RNAiFect Transfection Reagent (QIAGEN Inc., Valencia, CA, USA).

### Western blot analysis

Preparation of whole-cell lysates, cytoplasmic and nuclear extracts, and Western blot assay were performed as described previously [45, 47]. Equal amounts of protein from cell lysates were separated by SDS-PAGE and then transferred to PVDF membranes (Immobilon-P membrane; Millipore, Bedford, MA, USA). After blocking, the membranes were incubated with primary antibody overnight. The primary antibodies included the following: anti-PRL-3 (Abcam, Burlingame, CA, USA); anti-c-myc tag (Sigma-Aldrich, Deisenhofen, Germany); anti-EGFP (Clontech); anti-β-catenin (BD Biosciences Pharmingen, San Diego, CA, USA); anti-Slug (Santa Cruz Biotechnology Inc., Santa Cruz, CA, USA); anti-E-cadherin (BD Biosciences Pharmingen); anti-phospho-AKT (Ser473) (Millipore); anti-AKT (Cell Signaling Technology Inc., Danvers, MA, USA); anti-phospho-GSK (Ser9) (Cell Signaling); anti-GSK (Cell Signaling Technology Inc.); anti-phospho-ERK (Tyr204) (Santa Cruz Biotechnology); anti-ERK2 (Santa Cruz Biotechnology); anti-phospho-Src (Tyr418) (Invitrogen); anti-GAPDH (Invitrogen); anti-β-actin (Sigma-Aldrich); and anti-β-tubulin (Millipore). The membranes were then washed with PBST, followed by incubation with a horseradish peroxidase-conjugated secondary antibody (Santa Cruz Biotechnology Inc.). Bound antibody was detected using the Enhanced Chemiluminescence System (Amersham Biosciences, Piscataway, NJ, USA). Three independent experiments were performed.

### Subcellular localization of ectopically expressed PRL-3

To determine the subcellular localization of ectopically expressed PRL-3 in living cells, CL1-5 cells were transiently transfected with *pEGFP-PRL-3* or *pEGFP-C3* as a negative control. After 48 h, the cells were examined and imaged (1000×) using an Olympus BX51 epifluorescence microscope (Olympus Co., Tokyo, Japan).

### *In vitro* cell invasion assay

*In vitro* invasion assays were performed as previously described [48] using transwell chambers (8-μm pore size; Corning Costar, Cambridge, MA, USA) and transwell filters coated with Matrigel (R & D Systems GmbH, Wiesbaden, Germany). Cells (3 × 10^4^) were cultured on Matrigel and incubated overnight. The membranes were fixed with methanol and then stained with 20% Giemsa solution (Sigma Chemical, St. Louis, MO, USA). The total number of cells that invaded to the lower surface of the polycarbonate filter was counted under a light microscope (50× magnification).

### Migration assay

Cells (1 × 10^4^) were seeded in the upper compartment of transwell chambers (8-μm pore size). Medium supplemented with serum was added to the lower chambers. After 18 h, membranes were fixed and stained with Giemsa. The number of cells that migrated through the membrane to the lower compartment was counted.

### Colony-formation assay

To determine the level of anchorage-independent growth, 6-well plates were precoated with 0.7% agarose in PBS, and cells were seeded at 5 × 10^2^ cells per well in 0.35% agarose/RPMI-1640 with 10% FBS. The plates were incubated for 3 weeks and then stained with 0.5 mg/ml p-iodonitrotetrazolium violet. Colonies with a diameter > 1 mm were counted under an inverted microscope. For the anchorage-dependent growth assay, 500 cells per well were seeded in a 6-well plate, incubated for one week, and stained with 0.5% crystal violet. Colonies with a diameter > 1 mm were counted under an inverted microscope.

### *In vivo* tumorigenesis

Five-week-old nude mice (supplied by the National Laboratory Animal Center, Taipei, Taiwan) were maintained with autoclaved food and water. The PRL-3-transfected (PRL3-mixed and PRL3-18) or mock-transfected cells (1 × 10^6^) were injected subcutaneously into the dorsal region of nude mice (*n* = 6). Injected mice were examined every 2 or 5 days for the appearance of tumors, and tumor volumes were estimated from the length (*a*) and width (*b*) of the tumors, as measured by calipers, using the formula *V* = *ab*^2^/2 [49]. The mice developed tumors approximately 28 days after inoculation. The animal experiments were approved by the Institutional Animal Care and Use Committee of the National Chung Hsing University.

### Quantitative real time RT–PCR analyses

The expression level of the target gene was detected with SYBR Green real-time RT-PCR on an ABI Prism 7300 sequence detection system (Applied Biosystems, Grand Island, NY, USA), according to the manufacturer's instructions. The primers used for amplification of the target genes are listed in Supplementary Table S1. The expression of the target gene normalized to that of the TATA box-binding protein (TBP), which was used as the internal control, was defined as –ΔCT = – (CT_target_ – CT_TBP_), and the difference in the relative expression of the target gene between cell lines was calculated using the 2^−ΔΔCT^ method.

### Oligonucleotide microarray analysis

Total RNAs were isolated from PRL-3- and mock-transfected CL1-5 cells (stable pooled clones) using the RNABee reagent (Biogenesis, Inc., Poole, UK). cRNA preparation and array hybridization were performed according to the Human WG6 BeadChips Expression Analysis Technical Manual (Illumina, San Diego, CA, USA). The statistical analysis logic and algorithms used are described in the Illumina manual. After quantile normalization, genes with > 2-fold difference between PRL-3 and mock transfectants were subjected to pathway analysis using MetaCore software (GeneGo, St. Joseph, MI, USA). In addition, SYBR Green real-time RT-PCR was employed to confirm the results derived from the microarray analysis.

### Statistical analyses

All *in vitro* experiments were performed at least in triplicate. The data are presented as the mean ± standard deviation, and the significance of the differences was analyzed using an analysis of variance (ANOVA, Excel; Microsoft) or Student's *t*-test. All statistical testing was two-tailed, and values of *P* < 0.05 were considered significant.

## SUPPLEMENTARY MATERIALS TABLES AND FIGURES


