# Supplementary material for: Phosphatase of regenerating liver-3 inhibits invasiveness and proliferation in non-small cell lung cancer by regulating the epithelial-mesenchymal transition
Source: Oncotarget. 2016 Mar 8;7(16):21799–811. doi: 10.18632/oncotarget.7985 (PMC5008324; doi:10.18632/oncotarget.7985)
Supplement: Supplementary file 1 [file oncotarget-07-21799-s001.pdf]

Phosphatase of regenerating liver-3 inhibits invasiveness and proliferation in non-small cell lung cancer by regulating the epithelial-mesenchymal transition

Supplementary Materials

Supplementary Table S1: A list of genes modulated by PRL-3 and involved in the regulation of the epithelial-to-mesenchymal transition (EMT) pathway

GenBank UniGene	Symbol	Gene name	PRL3/Mock (Illumina)
Hs.360174	SNAI2	Snail homolog 2 (Slug)	0.07
Hs.490203	CALD1	Caldesmon 1	0.11
Hs.31595	CLDN1	Claudin-1	0.17
Hs.82028	TGFBR2	TGF-beta receptor type II	0.27
Hs.511899	EDN1	Endothelin-1	0.43
Hs.626544	JAG1	Jagged1	2.15
Hs.279594	TNFRSF1A	Tumor necrosis factor receptor superfamily, member 1A	2.26
Hs.414795	SERPINE1	Serpin peptidase inhibitor, clade E	2.44
Hs.464829	CDH2	N-cadherin	2.45
Hs.1976	PDGFB	Platelet-derived growth factor beta polypeptide	2.49
Hs.461086	CDH1	E-cadherin	3.06
Hs.512714	WNT7B	Wingless-type MMTV integration site family, member 7B	3.53
Hs.74615	PDGFRA	Platelet-derived growth factor receptor, alpha polypeptide	4.52

Supplementary Table S2: The primer list for real-time RT-PCR

Symbol	Forward primer	Reverse primer
PRL-3	5′-GGGCTCCAGTCCTTGTG-3′	5′-CGGTATTTCTCCAGGTAGG-3′
SNAI2	5′-GGCGCCCTGAAGATGCATAT-3′	5′-AAGGCTTCTCCCCCGTGTGA-3′
CDH1	5′-GGCCTGAAGTGACTCGTAACGA-3′	5′-TCAGCCGCTTTCAGATTTTCAT-3′
DKK1	5′-TTCCGAGGAGAAATTGAGGAAAC-3′	5′-TGACCGGAGACAAACAGAACCTT-3′
ANGPT2	5′-CTAACAGGAGGCTGGTGGTTTG-3′	5′-TCATCATGGTTGTGGCCTTGA-3′
AKAP12	5′- GACCATCACCATCACAGTTGGA -3′	5′- CTGTTCGATTATTTCGGGTGTCT -3′
TBP	5’-CACGAACCACGGGACTGATT-3’	5’-TTTTCTTGCTGCCAGTCTGGAC-3’

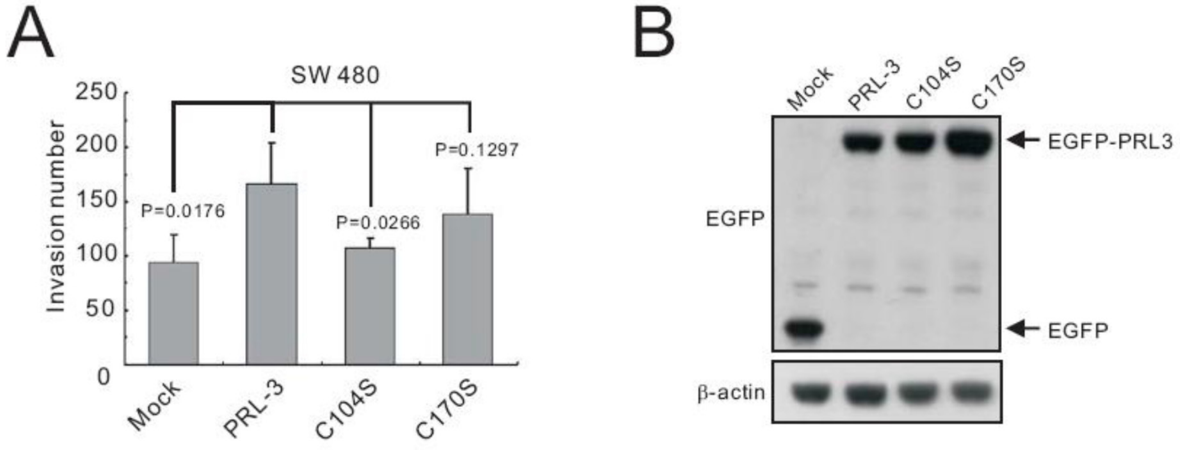

Supplementary Figure S1: Promotion of invasiveness in colon cancer cell line by PRL-3 overexpression. (**A**) Invasiveness ability of transient expression of wild-type PRL-3 and mutant PRL-3/C104S and PRL-3/C170S, as determined by Matrigel-coated transfilter invasion assay on SW480 colon cancer cells. The data are presented as the means ± S.D. of three experiments and the *P* value are compared with wild-type PRL-3 cells. (**B**) The ectopic expression of PRL-3 protein, as detected by Western blotting and EGFP antibody. b-actin was used as an internal control.

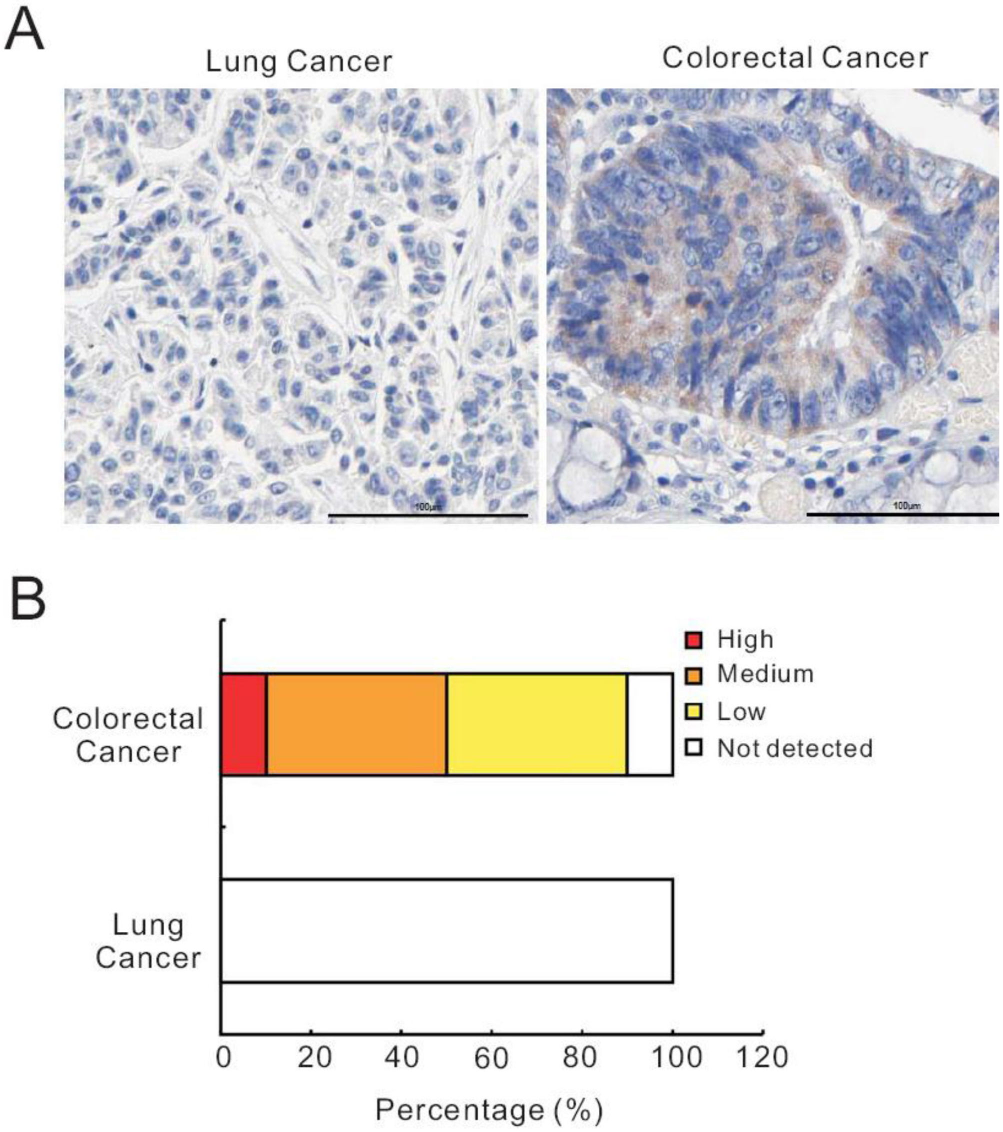

Supplementary Figure S2: PRL-3 expression in lung and colorectal cancer. The Human Protein Atlas database (www.proteinatlas.org) was used to determine the protein expression profiles of PRL-3 in human lung and colorectal cancer specimens. (**A**) Protein expression profiles are based on immunohistochemistry of human specimens. (**B**) The color determines the percentage of cancer samples in the database with a given expression level. The numbers of samples for each specimen are as follows: lung cancers (12) and colorectal cancers (10).

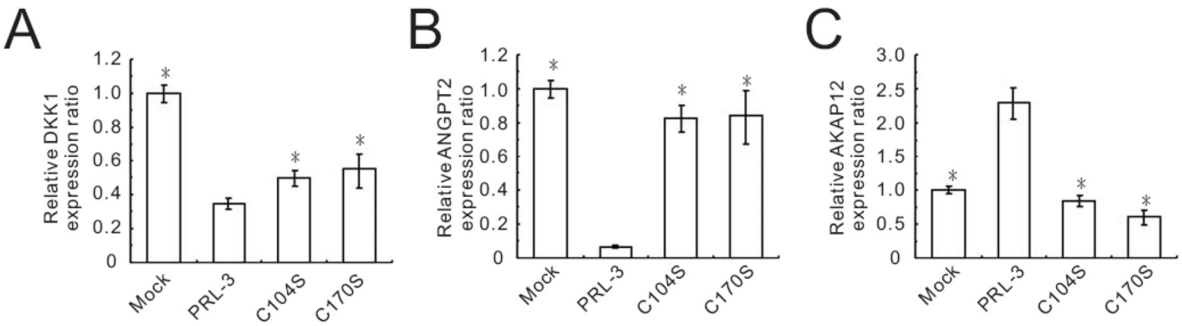

Supplementary Figure S3: PRL-3 expression regulates tumor-related genes. (**A**) *DKK1*, (**B**) *ANGPT2* and (**C**) *AKAP12* mRNA expression in wild-type and mutant PRL-3 transfectants, as detected by real-time RT-PCR. Stable CL1-5 transfectants expressing vector alone, wild-type PRL-3, mutant PRL3/C104S, or PRL3/C170S were employed in this study. The data are presented as the mean ± S.D. in triplicate. *$P < 0.05$, compared with the wild-type PRL-3 cells.
